# Co-Designing a Digital Platform for the Management of Modifiable Risk Factors and Comorbidities in Patients With Atrial Fibrillation: Mixed Methods Usability Study

**DOI:** 10.2196/95810

**Published:** 2026-10-22

**Authors:** Sofia Klavebäck, Frieder Braunschweig, Matthias Lidin, Emma Svennberg

**Affiliations:** 1 Department of Cardiology Heart and Vascular Center Karolinska University Hospital Stockholm Sweden; 2 Department of Medicine Huddinge Unit of Integrative Cardiovascular Research (ICCA) Karolinska Institutet Stockholm Sweden; 3 Department of Medicine Karolinska Institutet Solna, Stockholm Sweden

**Keywords:** atrial fibrillation, risk factors, mHealth, mobile health, person-centered care, digital health

## Abstract

**Background:**

Clinical guidelines recommend integrated, person-centered care with control of modifiable risk factors and coexisting conditions in patients with atrial fibrillation (AF), but many receive insufficient risk factor management. Digital health technologies may provide valuable support in addressing this gap.

**Objective:**

Our aim was to evaluate the usability of a co-designed digital platform for supporting person-centered management of modifiable risk factors in individuals with AF.

**Methods:**

This mixed methods usability study was conducted at a tertiary university hospital in Sweden between March and April 2025. Eligible participants with AF were recruited to evaluate the co-designed digital platform using the System Usability Scale (SUS) and semistructured interviews analyzed with inductive manifest content analysis.

**Results:**

A total of 22 patients were included (mean age 67.7, SD 7.6 y); 32% (7/22) were female, the mean BMI was 27.7 (SD 3.6) kg/m^2^, 65% (14/22) had paroxysmal AF, 36% (8/22) had persistent AF, and the median AF duration was 26 (IQR 13.3-72.8) months. Relevant comorbidities were hypertension (17/22, 77%), heart failure (8/22, 36%), diabetes mellitus type 2 (3/22, 14%), and ischemic heart disease (4/22, 18%). Usability was rated high, with a median SUS score of 78.8 (IQR 63.8-86.9), indicating above-average user acceptance. Participants’ requirements were summarized into 4 main categories and 10 subcategories. First, they valued a *clear layout*, *simple design,* and *easy navigation*. Second, they appreciated *positive content,* which is *informative*, *inclusive,* and *motivating*. Third, they requested *personalized information* on *different aspects* and provided on *different levels*. Fourth, they desired *individualized medical recommendations* that are *personalized* and *flexible* but *open to individual choice*.

**Conclusions:**

Participants found the usability acceptable and valued specifically a *clear layout*, *positive content*, *personalized information*, and *individualized medical recommendations*.

## Introduction

The incidence of atrial fibrillation (AF) increases with age and affects more than 3% of adults in a population-based Swedish study, making it the most common cardiac arrhythmia [1]. Patients can experience paroxysmal or persistent AF, and for symptomatic patients, guidelines recommend restoring sinus rhythm, which can be achieved by medical treatment, electrical cardioversion, or more permanently via catheter ablation [2].

AF is associated with nonmodifiable risk factors such as increasing age, height, and male sex [3]. However, it is also associated with modifiable and dynamic risk factors related to comorbidities including hypertension, hyperglycemia, obesity, obstructive sleep apnea, and mental health conditions, but also to unhealthy lifestyle choices including risk consumption of alcohol and physical inactivity [3]. Over half of AF cases in middle-aged adults can be attributed to inadequate control of modifiable risk factors [4]. Several studies have shown that better risk factor management is associated with lower risk of incident AF [5-7], sinus rhythm maintenance [8], and improved outcomes after catheter ablation [9]. Therefore, guidelines specifically recommend integrated management of modifiable risk factors for all persons with AF, especially those undergoing rhythm interventions [3]. Furthermore, the benefits of person-centered care have recently been emphasized, including multidisciplinary, coordinated, and tailored solutions focusing on shared decision-making [10].

Digital health interventions may facilitate person-centered care by supporting self-management and risk factor modification. Although such interventions have shown promising results in cardiovascular disease and other chronic conditions [11,12], evidence in AF is still limited. We therefore co-designed a digital platform to support person-centered management of modifiable risk factors in patients with AF where we now aim to evaluate usability.

Specifically, we aimed to investigate how patients with AF evaluate design, function, and user experience of a digital platform, to identify how to further optimize it according to the needs of the patients, the end users.

## Methods

### Description of the Co-Design Process

We co-designed a digital platform with the aim of improving support for patients with AF in the management of modifiable risk factors (Figure 1). As a first step, we conducted a study where we identified unmet needs with regard to modifiable risk factors among individuals with AF and further explored the specifics of the desired functions and usability of such a platform [13]. Specifically, participants with AF requested *person-centered information*, support in *managing healthy lifestyle habits*, and more *regular communication* with caregivers [13]. There was a general desire for person-centered care, defined as a partnership where interdisciplinary health care professionals actively listen to and involve the patient in forming a health plan together [14-17]. A prototype was developed based on these findings, tested with the same patient group, and subsequently adjusted according to their feedback, resulting in the co-designed digital prototype evaluated in this study. In this next step of the co-design, we aim to more broadly evaluate the usability of the prototype of our digital platform for supporting person-centered management of modifiable risk factors in individuals with AF.

**Figure 1:**
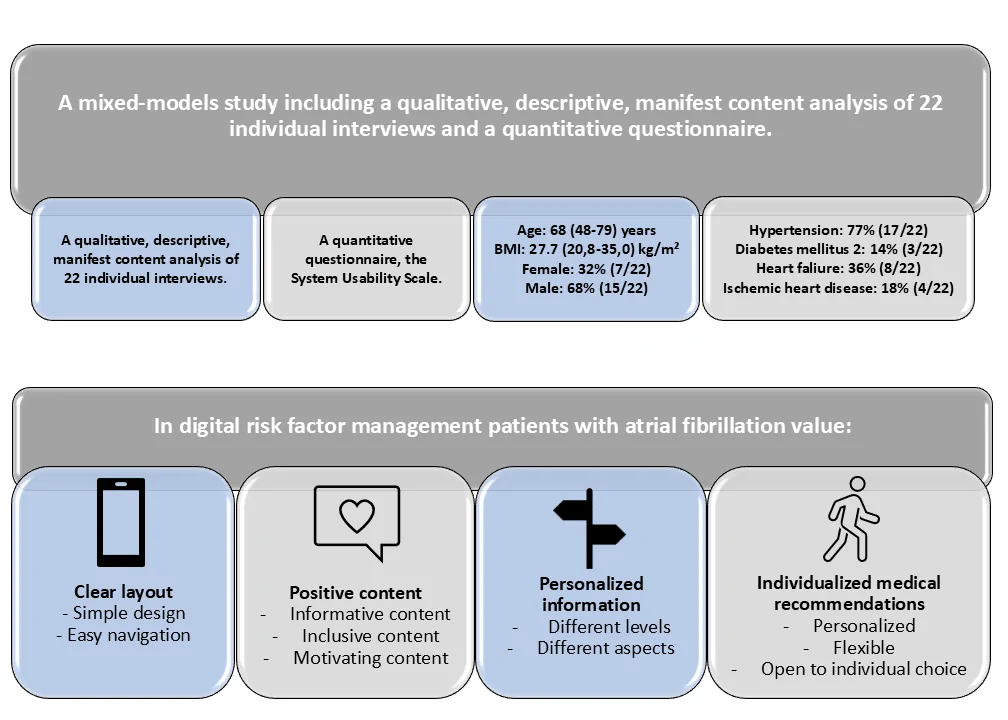
Graphical abstract.

### Description of the Digital Platform

The platform can be used on a smartphone or computer, where patients waiting for catheter ablation for AF enter their medical information, including data on comorbidities and medications, and answer questionnaires about symptoms and lifestyle. Based on their input, an algorithm identifies which factors are scored outside of targets recommended by the European Society of Cardiology guidelines for AF. For example, regarding comorbidities, if a patient enters a BMI>27 kg/m^2^ or blood pressure>130/80 mm/Hg, the algorithm identifies these possible targets to optimize based on guidelines. Questionnaires are used in a similar fashion to identify responses that might merit additional attention, for example, individuals who do not reach recommended targets of healthy eating habits or physical activity. These areas are then presented as suggestions to the user to improve. However, the choice of which recommendations to work with remains with the users. Based on the users’ choice, a week-long sprint is initiated, providing advice on their areas of improvement. These have been developed with a nurse, dietitian, physician, and physiotherapist and are targeted at each area of improvement with a new task each week. For example, the advice can include weight management support for elevated BMI, physical activity recommendations specific for patients with AF, and healthy recipes to improve eating habits. Users can also follow the progress of measurable values such as blood pressure and BMI over time. Examples of these interventions are illustrated in Figure 2 and Figure 3. Development is purely research-based, and there are no commercial interests involved.

**Figure 2:**
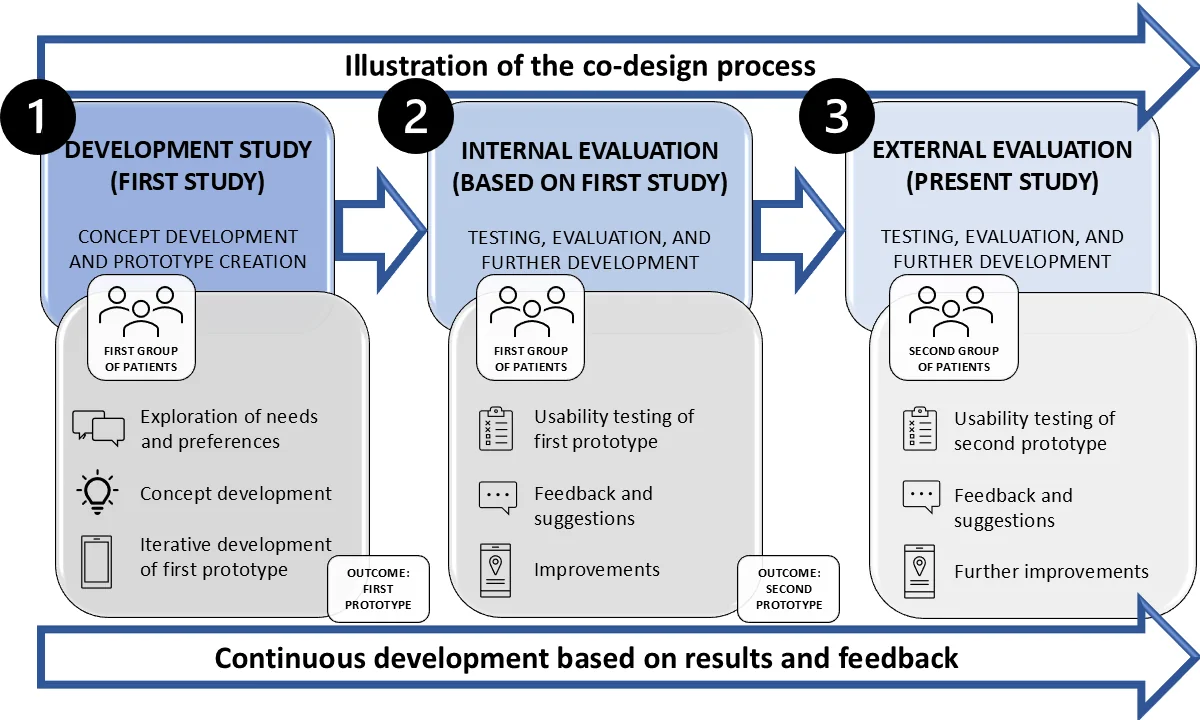
Illustration of the co-design process.

**Figure 3:**
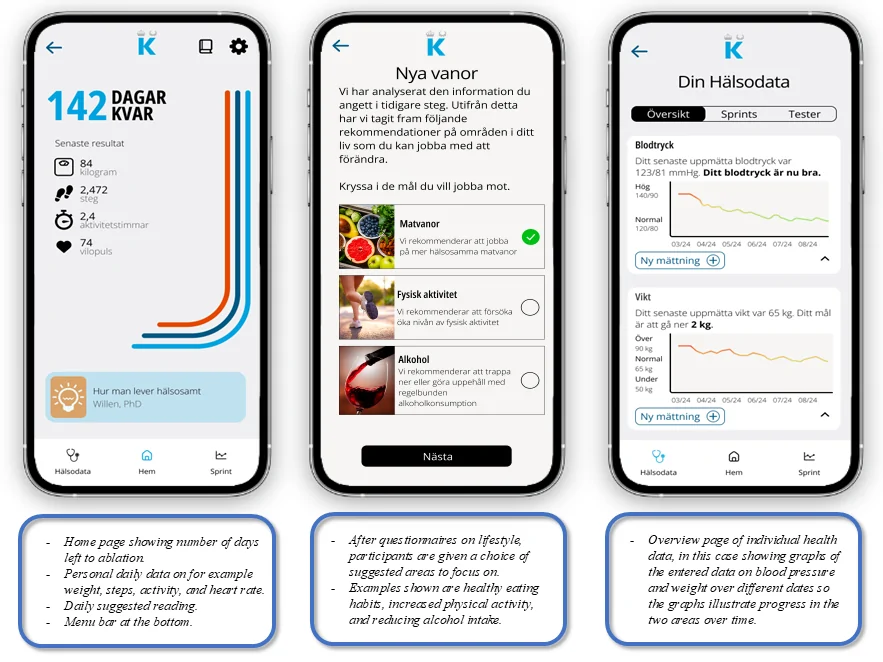
Illustration exemplifying our digital platform.

### Study Design

This was a mixed methods usability study including quantitative components to evaluate and rate our co-designed tool, as well as a qualitative component to further explore patients’ needs and perceptions and identify areas of improvement. In this study, the initial phase of the platform was tested, including signing up, answering questionnaires, and going through the suggested areas of improvement, and provided recommendations for feedback. First, a widely used quantitative scoring questionnaire for the usability of digital tools was used. Second, a qualitative, descriptive study was conducted with an inductive approach, based on semistructured individual interviews that were analyzed by manifest content analysis [18,19].

### Recruitment

Eligibility included patients with (1) a confirmed diagnosis of AF who were (2) admitted to the elective Cardiology ward at Karolinska University Hospital (Stockholm, Sweden), specifically for this condition during the interview period of 2 weeks (March 31, 2025, to April 11, 2025). The sole exclusion criterion was not being able to understand or speak Swedish. Eligible patients were identified among those admitted to the cardiology ward during the study period. They were approached in person by the first author (SK), who is a medical doctor and PhD student, and she had no prior relationship with any of the study participants. All participants first received oral and written information about the study; those who accepted participation were then asked to provide written informed consent. Signed forms were collected before data collection.

### Data Collection

Immediately after informed consent, first author SK collected baseline data from patients and medical records on age, sex, weight, BMI, medical conditions, as well as previous and ongoing treatments and medications. After this, SK left the room so that patients could begin the usability test alone with a study-provided smartphone with the prototype on the screen. They were suggested to take 1-2 hours to do this by themselves in their single-patient rooms at the cardiology ward but were able to call on the interviewer sooner if ready with the usability test or to request more time if needed. Most patients took about an hour for the usability test, and none demanded more time than 2 hours.

The testing procedure consisted of a structured usability test. Detailed instructions were given in writing to all participants (for exact translated instructions, refer to Multimedia Appendix 1) and included the following steps: creating a user, filling in questionnaires, choosing one of the recommended target areas suggested by the algorithm, and then going through the given recommendations within their suggested area, including, for example, requested measurements, activities, recipes, etc.

Immediately after testing, patients were first asked to answer a System Usability Scale (SUS) Questionnaire [20] (Multimedia Appendix 2). It is a widely used and well-known scoring system for the usability of digital tools consisting of 10 statements where participants answer each one on a Likert scale from 1 to 5 between “strongly disagree” and “strongly agree” [21]. Results are calculated to a total score between 0 and 100, where a higher score reflects a better perceived usability. Above 68 points is considered acceptable usability [21]. Second, directly after filling in the questionnaire, a semistructured individual interview was conducted by author SK, using a structured interview guide (Multimedia Appendix 3) covering topics of design, function, usability, and user experience.

### Data Analysis

Quantitative SUS data were analyzed using descriptive statistics, median (IQR). The interviews were recorded (audio), and a contracted transcription service was used to transcribe all interviews verbatim. The qualitative, descriptive, manifest content analysis with an inductive approach was performed according to Graneheim et al [18], Graneheim and Lundman [19], and Krippendorff [22]. The analysis was inductive, and categories emerged from the interview material. The transcripts were read several times and relevant meaning units were identified, condensed, and coded. Codes with similar content were then grouped into subcategories and broader categories. Examples of meaning units, codes, and categories are shown in Table 1. The analysis was conducted after completion of all interviews and included reading the transcribed interviews several times, identification of initial meaning units, condensation (reduction), labeling with codes, and creation of initial categories and subcategories. Wording was completely emergent from the interviews. Analysis was first done separately by author SK and author ML, who is a specialist nurse in cardiology and PhD and experienced in qualitative methods. The process was then further discussed with the author ES and FB, who are both electrophysiologists in the field of AF. They were presented with both raw and already coded material, and the developing categories and discussions were held to strengthen trustworthiness. Where disagreement, discussions were held until consensus was reached among all authors.

**Table 1:** Examples of meaning units and codes related categories.

Meaning units	Codes	Categories
…do not complicate it with many colors and arrows…	Simple design	Clear layout
…as easy and clear as possible to that you cannot go wrong …	Easy navigation	Clear layout
…inform without frightening…	Informative	Positive content
…non-binary, other…	Inclusive	Positive content
…it doesn’t have to be boring to be healthy…	Motivating	Positive content
…atrial fibrillation, what it is…	Different aspects	Personalized information
… with more for those who are extra interested…	Different levels	Personalized information
…depending on the patient…	Personalized	Individualized medical recommendations
…if one doesn’t want to, then one can just skip…	Flexible	Individualized medical recommendations
…one should be able to choose…	Open to individual choice	Individualized medical recommendations

### Ethical Considerations

The study protocol adhered to the Declaration of Helsinki and was approved by the Regional Ethical Review Board (Dnr 2025-01228-02). All participants were informed of the premises of the study beforehand, participation was voluntary, and they could at any time choose to discontinue their involvement in the study without having to state a reason for it. Data were transcribed anonymously and stored in a designated locked room at the Cardiology Research Department. A detailed COREQ (Consolidated Criteria for Reporting Qualitative Research) checklist is provided as a supplement (Multimedia Appendix 4).

## Results

### Participant Characteristics and Interviews

In total, 30 participants were eligible and approached. Out of 30 participants, 22 were included and 8 declined participation with the main reasons stated being lack of time and/or interest. The interviews of the 22 participants lasted, on average, 24.3 (SD 9.7) minutes each and resulted in a total interview time of 535.2 minutes with material for analysis. Baseline characteristics are presented in Table 2.

**Table 2:** Participant characteristics (N=22).

Characteristics				Participants
**Demographics**				
	**Age (y)**			
		Mean (SD)	67.7 (SD 7.6)	
		Median (IQR; range)	69 (62.25-72.75; 48-79)	
	**Sex**			
		Male, n (%)	15 (68.2)	
		Female, n (%)	7 (31.8)	
	Persistent, n (%)			8 (36.4)
	Paroxysmal, n (%)			14 (63.6)
	**Previous direct current cardioversions**			
		Mean (SD)	2.6 (4.2)	
		Median (IQR; range)	2 (0-3; 0-20)	
	**Previous ablations**			
		Mean (SD)	1.4 (0.6)	
		Median (IQR; range)	1 (1-2; 1-3)	
	**Duration of atrial fibrillation (months)**			
		Mean (SD)	49 (55.3)	
		Median (IQR; range)	26 (13.3-72.8; 6-216)	
**Risk factors**				
	**BMI (kg/m²)**			
		Mean (SD)	27.7 (SD 3.6)	
		Median (IQR; range)	27.9 (26.2-30.2; 20.8-35.0)	
	**CHADS-VA^a^ score**			
		Mean (SD)	2 (1.8)	
		Median (IQR; range)	1.5 (1-3; 0-8)	
	Hypertension, n (%)			17 (77.3)
	Diabetes, n (%)			3 (13.6)
	Heart failure, n (%)			8 (36.4)
	**Ejection fraction %**			
		Mean (SD)	49.8 (6.8)	
		Median (IQR; range)	50 (50-55; 30-60)	
	Ischemic heart disease, n (%)			4 (18.2)
	Currently smoking, n (%)			0 (0)
	Ceased smoking, n (%)			13 (59.1)
	Consumes alcohol, n (%)			9 (40.9)
	**Alcohol standard glasses per week**			
		Mean (SD)	2.4 (4.2)	
		Median (IQR; range)	0 (0-1; 0-18)	
**Treatment, n (%)**				
	Betablockers			19 (86.4)
	Angiotensin-receptor neprilysin inhibitors / Angiotensin-converting-enzyme inhibitors			16 (72.7)
	Mineralocorticoid receptor antagonists			7 (31.8)
	Calcium inhibitors			3 (13.6)
	Statins			15 (68)
	Antiarrhythmics			6 (27.3)
	Non-vitamin K oral anticoagulants			21 (95.5)
	Warfarin			1 (4.5)

^a^CHADS-VA: risk score including congestive heart failure, hypertension, age, diabetes mellitus, stroke, transient ischemic attack, thromboembolism, and vascular disease.

### Quantitative Results – System Usability Scale

The SUS questionnaire resulted in a median score of 78.8 (IQR 63.8-86.9) points, presented in Table 3.

**Table 3:** Results: System Usability Scale scores by item and total.

System Usability Scale Score	Median (IQR)
I think that I would like to use this system frequently	4 (3.5-5)
I found the system unnecessarily complex	1 (1-2)
I thought the system was easy to use	4 (4-5)
I think that I would need the support of a technical person to be able to use this system	1 (1-2)
I found the various functions in this system were well integrated	4 (3-5)
I thought there was too much inconsistency in this system	2 (1-3)
I would imagine that most people would learn to use this system very quickly	4 (3-5)
I found the system very cumbersome to use	2 (1-2.5)
I felt very confident using the system	4 (3-5)
I needed to learn a lot of things before I could get going with this system	2 (1-3)
Total score	78.8 (63.8-86.9)

Responses were relatively consistent across the individual SUS items, with median scores ranging from 1 (IQR 1-2) to 4 (IQR 4-5) on the 0-5 scale. One participant indicated responses between 2 response options on the 1-5 Likert scale; these responses were recorded as the corresponding midpoint values (eg, 1.5 or 3.5). In exploratory subgroup analyses, the median SUS score was 85.0 (IQR 75.0-95.0) among female participants and 72.5 (IQR 60.0-83.8) among male participants. Participants aged <70 years had a median SUS score of 80.0 (IQR 71.3-86.3), compared with 76.3 (IQR 49.4-86.9) among those aged ≥70 years. These analyses were descriptive, and no statistical testing was performed. The 2 participants with the highest SUS scores gave all items the best possible rating. However, the 2 participants with the lowest SUS scores also expressed during the subsequent qualitative interviews that they found the platform unnecessarily complex and cumbersome and that they would need to learn more before using it.

### Qualitative Results—Categories

#### Overview

Some patients made initial comments regarding the developed digital tool:

…It is so good that you are working on this type of tool.

…I would absolutely want to use this.

…This app, I think it can be meaningful, because the wait is long and you have a lot of questions.

The qualitative analysis of interviews revealed four main categories and 10 subcategories related to participants’ preferences and perceptions, and these are summarized in Textbox 1.

**Results: categories and subcategories.**

**Clear layout**

- Simple design
- Easy navigation

**Positive content**

- Informative
- Inclusive
- Motivating

**Personalized information**

- Different aspects
- Different levels

**Individualized medical recommendations**

- Personalized
- Flexible
- Individual choice

First, they value a *clear layout* with *simple design* and *easy navigation*. Second, they appreciate *positive content,* which is *informative*, *inclusive,* and *motivating*. Third, they request *personalized information* on *different aspects* and provided on *different levels*. Fourth, they request *individualized medical recommendations* that are *personalized* and *flexible* but *open to individual choice*.

#### Clear Layout

The first category, a *clear layout,* was consistently emphasized throughout the interviews and included the subcategories *simple design* and *easy navigation*.

Participants expressed that it was a digital tool with a *simple design,* which is more likely to be understood and used by users of different ages and different backgrounds. One participant noted that:

I like that it is simple and not too many different colors, because often diagrams and such can become too much with different colors and a little difficult to read. I like this simplicity, and the text is a good size.

Frequently brought up was that using different colors, texts, and figures can become cluttered and be frustrating to users. One individual summarized that:

It is good, it is simple, it is easy to read. Do not complicate it with many colors and arrows, it is not necessary. I think that most appreciate simplicity.

Participants agreed that a simple design, without unnecessary complexity, is essential in a digital tool they would see themselves using over time.

One issue repeatedly raised by participants was the importance of *easy navigation* as an important determinant to further improve satisfaction with the digital tool. As one individual suggested:

You should be able to go back in an easy way, a go-back button, you should try to make this intuitive.

It was clear amongst participants that ease of navigation is essential to facilitate and avoid unnecessary frustration for users.

#### Positive Content

The second category, the desire for *positive content,* recurred throughout interviews and included the subcategories *informative*, *inclusive,* and *motivating* content.

Central for participants was that the content provided should focus on being *informative*. It was agreed that informative content is appreciated, but that it is not received well if presented as too demanding or alarming. As 1 participant pointed out about our information:

It is an important balance, so that you do not scare people. Inform without frightening.

Information also needs to be adapted to the intended users, as 1 participant mentioned:

Not in medical language.

This highlights how the overall tone of the content needs to be informative, yet accessible to be received positively by users.

Participants also valued our content that was perceived as *inclusive* to individuals regardless of age, gender, economic, and social situation. One participant noted and appreciated:

Do you have non-binary or other? Yes, you do.

Some brought up that the recommendations provided on healthy eating habits need to also be inclusive of individuals with different preferences and lifestyles, for example, regarding eating habits to include also:

What is gluten-free and vegetarian.

The findings underscore the importance of framing information in a way respecting diversity.

It was agreed that content needs to be *motivating* to the intended users. Many mentioned how motivating content is perceived as more respectful, and that motivation is much more likely to have an effect as compared with shaming or lecturing users. One participant emphasized that it is essential to encourage and inspire users and convey:

That it should be joyful as well, it does not have to be boring to be healthy.

This demonstrates how essential it is to present information in a way that is motivating and respectful to users to empower them.

#### Personalized Information

The third category, *personalized information,* emerged and included the subcategories of personalizing information on *different aspects* and providing it on *different levels*.

Participants expressed a desire for personalized information on *different aspects* tailored to their individual needs. Some participants wanted basic information on, for example:

What it is that you do during an ablation. And concerning atrial fibrillation, what it is.

Others requested in-depth information on associated topics such as causes, risk factors, genetics, mechanisms, medication, interactions, exercise, psychological effects, and effects on sexual function. Some suggested having links or icons leading to more detailed information.

This highlights the need for a wide variety of information on different aspects but personalized to support both users with limited prior knowledge and those seeking more detailed understanding.

The importance of information at *different levels* was another need that came up consistently in all the interviews. Some participants felt that they wanted only general information, while others felt that they had already had a lot of information on AF.

Some participants wanted much more and in-depth information provided, such as:

You could maybe press a button for references, for the best studies and meta-analyses.

Thus, having access to information on different levels is essential.

#### Individualized Medical Recommendations

The fourth central category was the desire for *individualized medical recommendations*, which included the subcategories of *personalized* and *flexible* but *open to individual choice*.

An important feature requested by participants was that medical recommendations should be *personalized*. Participants brought up the heterogeneity in the group of individuals with AF, with a large range of knowledge, engagement, motivation, and different lifestyles. It was mentioned how frustrating it is to get recommendations to stop smoking and reduce alcohol consumption to individuals who stopped smoking and drinking years ago. Some felt frustrated getting recommendations on physical activity that they could not perform due to other diagnoses and disabilities. There was general agreement that the recommendations provided should be personalized.

Participants also expressed a desire for *flexibility* in the medical recommendations provided. There should be no shame in some not being able to perform a certain recommendation, and as 1 participant suggested, they should be able to choose digitally from recommendations they felt were appropriate, such as:

Today I choose… click… a 20-minute walk.

This underlines the importance of being flexible in recommendations so as not to lose user interest and involvement.

Also central in the interviews was the importance of recommendations being open to *individual choice*. Most participants requested some level of individual choice in different terms, and many pointed out how each patient is an individual with their own preferences:

People react to information in different ways, and what one may think is good, another might feel this is not something I want.

They emphasized the desire not to feel mastered but to feel respected as adults and to feel like they had a choice and would be supported regardless. This reflects a preference for personalized, nonprescriptive guidance that accommodates individual needs and circumstances.

## Discussion

### Principal Results

In this study, participants reported overall satisfaction with our developed digital tool. The SUS score is a validated measure of perceived usability [23]. Our digital tool achieved a median SUS score of 78.8 (IQR 63.8-86.9). The fact that the 2 lowest scorers found it to be unnecessarily complex and cumbersome, and that they would need to learn more before using it, aligned with the findings in the qualitative analysis, which revealed that persons with AF particularly valued a *clear layout, positive content, personalized information*, and *individualized medical recommendations*. The quantitative and qualitative findings complemented one another, with the SUS indicating overall acceptable usability while the interviews identified specific design features that influenced participants’ experiences and highlighted priorities for further development.

The layout emerged as central to determining our participants’ satisfaction with the digital tool. Regardless of the content, people may be less likely to use a digital tool if considered too difficult and complex to use. For example, a previous meta-analysis on 114 digital health apps found that apps with a higher SUS score were more likely to be adopted and used consistently [21]. The same meta-analysis aimed to investigate whether the SUS score could be used for assessing usability and found it to be reliable and that a mean score above 68 was acceptable [21]. In our interviews, participants appreciated simple layouts, and this was strengthened by the overall median SUS score of 78.8 (IQR 63.8-86.9), which confirmed the acceptability to users. Furthermore, it is important to consider the demographics of the intended users, who, in the case of individuals with AF, tend to be slightly older; in our study, the age range was 48-79 years. A systematic review of 132 studies, for a population older than 60 years of age, demonstrated that simplified interfaces, clear navigation, and readable fonts were especially important to this group [24]. Because of the age range of users, it is essential to clarify and simplify layout, design, and navigation in health apps.

Maintaining an informative, inclusive, and motivating tone throughout the content provided was crucial to the participants in this study. This is in line with findings in our previous study where participants requested information conveyed in an appropriate manner without being condescending or moralizing [13]. The participants valued communication that was inspiring and motivating [13]. This theme has been recognized by other authors, for example, in a review of digital weight management interventions where regular and encouraging feedback was appreciated by participants requesting that the tone should not be too generic or impersonal [25]. In a review on lifestyle management intervention, it was identified that customized and personalized feedback was one of the features responsible for successful implementation of digital interventions [26], highlighting the need for positive and motivating content.

Personalized information was central to our participants, which relates to the concept of person-centered care, where the patient is an active part in jointly planning individualized care [14]. Similarly, in our prior study, participants specifically desired person-centered and customized information [13]. In both studies, they wished for information about many different aspects of AF, such as healthy lifestyle, eating habits, physical activity, alcohol consumption habits, stress, and medication [13]. Participants in both studies also requested information to be readily available but at different levels, so as to fit each individual’s backgrounds, prior knowledge, and preferences [13]. Another study evaluated the “INFORM-AF” program, a co-designed digital AF educational program developed by Ferguson et al and delivered to participants’ smartphones via the QStream platform, and they reported an excellent SUS score of 84 [27,28]. This was higher than in our study, demonstrating that the present digital platform has room for improvement in terms of usability.

Regarding the medical recommendations given, personalization and flexibility emerged as central to users. In cardiovascular medicine, there is a need for a shift toward multidisciplinary, coordinated, and tailored solutions focusing on shared decision-making [10]. To achieve person-centered care, an essential component is “respecting the patient’s information needs and preferences” [29], which could mean integrating tailored information with shared decision-making. Tailored information was also requested by participants in our previous study [13]. Furthermore, a review of digital weight management interventions concluded that personalization is an essential component for the success of the intervention [25]. Additionally, a systematic review of the literature on mobile health (mHealth) apps for managing AF found that an app for tailored patient education rated positively on all aspects [30]. The mAFA app, developed by Guo et al [31], includes clinical decision-support tools with educational materials, patient involvement strategies with self-protocols and structured follow-up [31]. It was found to be easy, user-friendly, and helpful, and resulted in increased knowledge of AF, improved drug adherence and satisfaction, and quality of life scores [31]. It was also associated with a lower rate of the composite outcome (ischemic stroke, systemic thromboembolism, death, and rehospitalization), and lower rates of rehospitalization [32]. These findings highlight the potential of digital health intervention to improve outcomes, which warrants further research.

The digital platform used in this trial has been co-designed with a group of patients with AF who were initially interviewed for requests and requirements of a digital platform for lifestyle and risk factor management. [13] The same individuals were then asked to test the platform further and provide their feedback on further development. In our current trial, we have included patients who were not part of the original development to test the digital platform. From this trial, we will set out to further develop the platform, focusing particularly on navigation, simplicity, layout, and personalization. The co-design process shaped the platform by turning patient feedback into design changes, especially a simpler interface, clearer navigation, and less visual clutter. Patients also pushed for more control and less directive advice, which led to a choice-based system with layered information at different levels. These decisions likely improved usability and acceptability in the findings by making the tool easier to use and more aligned with how patients want to engage. Many recommendations for managing comorbidities and healthy lifestyle habits for patients with AF are similar to those for other patient groups. However, this study highlights that the unpredictable nature of AF, the wide range of functional impact, and its association with psychological and physical stress and reduced quality of life require flexible, individualized support for patients with AF.

Trustworthiness of a qualitative study can be evaluated through credibility, transferability, dependability, confirmability, and authenticity [18,19]. This study demonstrates high credibility, as the participants had relevant experience with AF and risk factors. Transferability is good as baseline characteristics are representative of individuals with AF undergoing intervention. There were no current smokers in the study population, which is consistent with the low prevalence of smoking in Sweden. However, this may limit the generalizability of the findings to individuals who smoke. To maintain dependability, confirmability, and authenticity, coding was done independently, and then ongoing discussions were held throughout the analysis process until consensus was reached. In this study, we support the conclusion by providing citations supporting the results and analysis.

### Limitations

Although the results are likely generalizable to persons with AF as reflected by the baseline characteristics (Table 2), a main limitation is that all participants were recruited from a single Swedish tertiary hospital with a large AF ablation service, which may reduce generalizability. It is worth noting that they were hospitalized specifically for AF, and this might create a selection bias of patients who have a slightly better understanding of AF in general. Although the SUS score is widely used and recommended for scoring perceived usability [23], it has inherent limitations, as it yields a single overall usability score without identifying domain-specific problems or offering qualitative insights, since it does not allow participants to elaborate on their responses. To address this, we also performed a qualitative analysis using a structured interview guide designed with open-ended questions to encourage participants to speak freely, to allow for a person-centered exploration of themes, perceptions, and meaning, and thereby reduce the risk of bias. Furthermore, the results were independently coded by 2 of the authors (SK and ML), and any disagreement was discussed multidisciplinary until consensus was reached among all authors. Nevertheless, no repeat interviews, field notes, or member checking were performed, and some risk of subjective interpretation and potential bias remains and cannot be fully excluded

As generative AI becomes more widely available, disease-specific platforms might complement these tools by providing individualized, guideline-based support integrated with clinical care. These findings will guide further development of digital tools for better risk factor management for individuals with AF undergoing intervention. Future testing is planned to be performed at home in a real-world setting, and we also aim to include other AF groups than those hospitalized. In addition, we will also aim to make sure we have a representative sample of older patients, and individuals with lower digital literacy. Future research should assess the effectiveness of digital tools in promoting better management of risk factors in AF, improved outcomes after AF intervention, and better utilization of resources.

### Conclusions

Persons with AF desire support in managing modifiable risk factors and find it likely that a digital tool could be used to facilitate this. In our digital tool for risk factor management, participants valued specifically a *clear layout, positive content, personalized information*, and *individualized medical recommendations*. This feedback will be used to guide development and guide work to develop digitally aided and person-centered management of modifiable risk factors for persons with AF, hopefully improving outcomes and reducing the cost of care.

## Data Availability

Not available due to ethical regulations.

## Appendix

Multimedia Appendix 1 Usability test.

Multimedia Appendix 2 System Usability Scale.

Multimedia Appendix 3 Interview guide.

Multimedia Appendix 4 COREQ (Consolidated Criteria for Reporting Qualitative Research).

## Abbreviations

AF: atrial fibrillation
COREQ: Consolidated Criteria for Reporting Qualitative Research
mHealth: mobile health
SUS: System Usability Scale
