# Supplementary material for: Co-Designing a Digital Platform for the Management of Modifiable Risk Factors and Comorbidities in Patients With Atrial Fibrillation: Mixed Methods Usability Study
Source: JMIR Hum Factors. 2026 Oct 22;13:e95810. doi: 10.2196/95810 (PMC13740813; doi:10.2196/95810)
Supplement: Supplementary file 1 [file humanfactors_v13i1e95810_app1.docx]

Usability test

WAIT-App

# Goal

To evaluate the prototype to give feedback on design, usability, functions.

# Testing

You will now create a test-user but should not use your own name or personal data.

| **Login** | |
| --- | --- |
| **Task** | Create a user with a fake name and password (1234). |
| **Questions** | Design? Ease of use? Preference? (bank-ID, e-mail, etc) |
| **Registration** | |
| **Task** | Fill in data with fake numbers (height 170 cm, weight 80 kg, etc). |
| **Questions** | Design? Ease of use? Understandable parameters? Missing? |
| **Questionnairs** | |
| **Task** | Fill in questionnaires with fake responses. |
| **Questions** | Design? Ease of use? Understandable questions? |
| **Focusareas** \|; **Task** \| Choose an area of focus (physical activity/nutrition) and look through.; **Questions** \| Areas? Content? Recommendations? Suggestions?; **Measurements** \|; **Task** \| Go to your data and add additional fake values and view results.; **Questions** \| Design? Ease of use? How do you prefer to visualize data?; **Recipes** \|; **Task** \| Go to recipes and look through.; **Questions** \| Design? Ease of use? Preference? | |
