# Supplementary material for: Co-Designing a Digital Platform for the Management of Modifiable Risk Factors and Comorbidities in Patients With Atrial Fibrillation: Mixed Methods Usability Study
Source: JMIR Hum Factors. 2026 Oct 22;13:e95810. doi: 10.2196/95810 (PMC13740813; doi:10.2196/95810)
Supplement: Supplementary file 2 [file humanfactors_v13i1e95810_app2.docx]

**Usability of WAIT-app**

**1. I would like to use the WAIT-app.**

________________________________________________

1	2	3	4	5

Strongly disagree	Strongly agree

**2. I found the WAIT-app unecessarily complex.**

________________________________________________

1	2	3	4	5

Strongly disagree	Strongly agree

**3. I found the WAIT-app easy to use.**

________________________________________________

1	2	3	4	5

Strongly disagree	Strongly agree

**4. I would need the support of a technical person to be able to use the WAIT-app.**

________________________________________________

1	2	3	4	5

Strongly disagree	Strongly agree

**5. I think the various functions in the WAIT-app were well integrated.**

________________________________________________

1	2	3	4	5

Strongly disagree	Strongly agree

**6. I found too much inconsistency in the WAIT-app.**

________________________________________________

1	2	3	4	5

Strongly disagree	Strongly agree

**7. I would imagine that most people would learn to use the WAIT-app very quickly.**

________________________________________________

1	2	3	4	5

Strongly disagree	Strongly agree

**8. I found the WAIT-app very cumbersome to use.**

_______________________________________________

1	2	3	4	5

Strongly disagree	Strongly agree

**9. I feel very confident in using the WAIT-app.**

_______________________________________________

1	2	3	4	5

Strongly disagree	Strongly agree

**10. I needed to learn a lot of things before I could get started with the WAIT-app.**

________________________________________________

1	2	3	4	5

Strongly disagree	Strongly agree
