# Supplementary material for: Co-Designing a Digital Platform for the Management of Modifiable Risk Factors and Comorbidities in Patients With Atrial Fibrillation: Mixed Methods Usability Study
Source: JMIR Hum Factors. 2026 Oct 22;13:e95810. doi: 10.2196/95810 (PMC13740813; doi:10.2196/95810)
Supplement: Supplementary file 3 [file humanfactors_v13i1e95810_app3.docx]

**Interview-guide for evaluation of the WAIT prototype of smartphone application**

Introduction – Presentation and aim

1. How do you perceive the functionality of the WAIT-app?

2. What do you think about the design of the WAIT-app?

3. How often and when would you use the WAIT-app in your daily life?

4. Do you think the WAIT-app would help you achieve your goals?

5. Which goals do you believe the WAIT-app could help you achieve?

6. Are there any functions that you perceive as unnecessary in the WAIT-app?

7. Are there any functions that you perceive are missing in the WAIT-app?

8. Which parts of the WAIT-app would you use the most?

9. Which parts of the WAIT-app would you use the least?

10. Would you recommend the WAIT-app to others in a similar situation?

11. Would you like to continue using the WAIT-app?

12. What specifically would you like us to change in the WAIT-app?

Follow-up questions related to the above discussion.

Conclusion – Summary and thank you.
